# Impact of Vitamin D Supplementation on Hospitalizations for Infection: Results of the D-Health Trial Revisited

**DOI:** 10.3390/nu18142276

**Published:** 2026-07-11

**Authors:** Youqing Wang, Sha Sha, Tafirenyika Gwenzi, Ben Schöttker, Hermann Brenner

**Affiliations:** 1 Cancer Prevention Graduate School, German Cancer Research Center (DKFZ), Im Neuenheimer Feld 280, 69120 Heidelberg, Germany; youqing.wang@dkfz-heidelberg.de; 2 Faculty of Medicine, University of Heidelberg, 69115 Heidelberg, Germany; 3 Department of Cancer Prevention, Zhejiang Cancer Hospital, Hangzhou 310022, China; 4 Division of Clinical Epidemiology of Early Cancer Detection, German Cancer Research Center (DKFZ), 69120 Heidelberg, Germany; 5 Network Aging Research, University of Heidelberg, 69120 Heidelberg, Germany

**Keywords:** vitamin D, randomized controlled trials, cohort study, infection, hospitalization

## Abstract

Background: The D-Health Trial, by far the largest trial investigating the impact of vitamin D supplementation on infection-related hospitalizations, did not find any protective effects. However, this trial was conducted in a mostly vitamin D-replete population of older adults from Australia. Objectives: We aimed to derive trial results that would have been expected if the trial had been conducted in a vitamin D-insufficient or -deficient population. Methods: Our analyses are based on data from the UK Biobank cohort. Participants meeting the D-Health Trial eligibility criteria (*n* = 185,809) were either weighted to match the trial’s baseline 25-hydroxy-vitamin D (25(OH)D) distribution (mean 77 nmol/L [30.8 ng/mL]) or restricted to those with insufficiency or deficiency (25(OH)D < 50 nmol/L [<20 ng/mL] or <30 nmol/L [<12 ng/mL], respectively). A 38 nmol/L (15.2 ng/mL) increase in 25(OH)D was assumed, as observed in the trial. Infection-related hospitalizations were identified via International Classification of Diseases, 10th Revision (ICD-10) codes. Incidence rate ratios (IRRs), adjusted for a comprehensive list of potential confounders, were estimated using negative binomial models over a follow-up period of 5.7 years, corresponding to the median follow-up time of the D-Health Trial. Results: In analyses reflecting the vitamin D-replete trial population, no protective association was observed (IRR 1.08, 95% CI 0.99–1.17), consistent with the reported trial results. In contrast, among participants with baseline 25(OH)D < 50 nmol/L, a 38-nmol/L increase in 25(OH)D was associated with lower risks of hospitalization for any infection (IRR 0.85, 95% CI 0.80–0.90), with even stronger associations for participants with baseline 25(OH)D < 30 nmol/L (IRR 0.79, 95% CI 0.73–0.86). Findings were consistent across infection types, sex, and body mass index (BMI). Conclusions: Null findings in the vitamin D-replete D-Health trial population were to be expected. Substantial protective effects might have been expected in vitamin D-insufficient or -deficient populations.

## 1. Introduction

Vitamin D has long been recognised for its role in calcium homeostasis, but increasing evidence suggests that it also plays an important role in immune regulation [1,2,3]. Vitamin D receptors and the enzyme 1α-hydroxylase, which converts circulating 25-hydroxyvitamin D [25(OH)D] into its active form, calcitriol, are expressed in several immune cell types, including macrophages, monocytes, dendritic cells, and lymphocytes. This allows immune cells to locally activate vitamin D and respond to it in an autocrine or paracrine manner. Experimental studies have shown that calcitriol can enhance innate immune defence by inducing antimicrobial peptides, particularly cathelicidin and defensins, which contribute to the elimination of bacteria, viruses, and other pathogens [4,5]. In addition, vitamin D may modulate inflammatory responses by reducing the production of pro-inflammatory cytokines and supporting a more regulated immune response [5,6,7].

Consistent with these biological mechanisms, observational studies have frequently reported associations between low circulating 25(OH)D concentrations and increased risks of respiratory infections and infection-related hospitalizations [8,9,10,11,12,13,14]. Analyses from NHANES III showed that lower serum 25(OH)D concentrations were associated with a significantly higher prevalence of recent upper respiratory tract infections [8]. In a cohort of 1713 Medicare beneficiaries, serum 25(OH)D concentrations below 37.5 nmol/L were associated with a 2.8-fold higher risk of hospitalization with infection (95% CI 1.3–5.9) [12]. Lower vitamin D concentrations have also been associated with an increased incidence of acute viral respiratory tract infections in healthy adults [11] and with increased risks of lower respiratory tract infections and infection-related hospitalization among newborns, infants and children [9,10,14].

Given the high prevalence of vitamin D insufficiency and deficiency, which are commonly defined by 25(OH)D concentrations <50 and <30 nmol/L, respectively [15], among older adults in many parts of the world [16,17,18], it has been hypothesized that vitamin D supplementation might be an effective and cost-effective approach to lower the heavy burden of disease due to infections.

However, findings from clinical trials of vitamin D supplementation for infection prevention have been inconsistent. Some meta-analyses have suggested modest protective effects [19,20], while some reported no clinical effects [21,22]. A recent post hoc analysis of by far the largest clinical trial reporting on the effects of vitamin D supplementation of hospitalizations due to infection, the D-Health Trial in Australia, which included 21,315 adults aged 60–84 years, did not find a protective effect of monthly oral vitamin D3 supplementation with 60,000 IU vitamin D [23]. These findings have been commonly interpreted as evidence that vitamin D supplementation does not reduce infection-related hospitalizations.

A potential explanation for the inconsistent trial findings is that some of the largest supplementation trials were conducted in populations with a relatively high baseline vitamin D status. In the D-Health trial, the mean baseline 25(OH)D concentration was approximately 77 nmol/L, and only a small minority of 13% of participants had vitamin D deficiency or insufficiency [23]. Trials conducted predominantly in vitamin D-replete populations may have limited ability to detect benefits that are confined to individuals with low baseline 25(OH)D concentrations. This highlights the importance of considering baseline vitamin D status when interpreting the trial results.

We recently used UK Biobank data to examine expected effects of vitamin D supplementation on all-cause mortality [24], showing that major benefits would be expected among individuals with vitamin D insufficiency or deficiency, whereas null results were to be expected in trials conducted in vitamin D-replete populations, such as the VITAL trial [25] or the D-Health trial [23].

Here, we applied an analogous approach to assess and compare the expected impact of vitamin supplementation on hospitalizations due to infection when either weighting the study population to match the baseline 25(OH)D distribution observed in the D-Health trial or when focusing the intervention on people with vitamin D insufficiency or deficiency.

## 2. Methods

### 2.1. Study Population and Data

Our analyses used data from the UK Biobank, a large prospective cohort established between 2006 and 2010 that enrolled over 500,000 individuals aged 40–69 years from across the United Kingdom [26]. Baseline data were collected at 22 assessment centres in England, Scotland, and Wales through a combination of interviews, touchscreen questionnaires, and standardized physical and medical examinations [26]. Hospital Episode Statistics (HESs) have been linked to the UK Biobank. HESs are national data for England that contain ICD-10 diagnoses for hospital inpatient admissions [27]. All participants gave written informed consent. The study was conducted according to the guidelines of the Declaration of Helsinki and was approved by the North West Haydock Research Ethics Committee (#16/NW/0274, 13 May 2016).

### 2.2. Exposures, Outcomes and Covariates

The exposure was defined as an increase in mean serum 25(OH)D concentration by 38 nmol/L, corresponding to the difference in 25(OH)D concentrations observed between subsamples of the intervention group and the placebo group at the end of follow-up in the D-Health study [28]. This value was selected because it reflects the average increase in serum 25(OH)D concentration achieved by vitamin D supplementation in the D-Health Trial and therefore provides a clinically relevant increase for comparison with the trial findings.

The primary outcome was hospitalization for infection, defined as the total number of hospital admission episodes with a principal diagnosis code indicating infection. In the UKB database, the first date of in-patient diagnosis for each ICD-10 code was extracted. The infection-related hospitalizations were identified based on principal diagnosis ICD-10 codes A00–B99, J00–J22, and L00–L08. The secondary outcomes included hospitalizations for specific infection types, namely respiratory tract infections (RTIs) (J00–J22), gastrointestinal infections (GIs) (A00–A09), and skin infections (SIs) (L00–L08).

A comprehensive set of potential confounders was considered as covariates, including age, sex, education, socioeconomic deprivation, lifestyle behaviours, sun exposure variables, and dietary factors. Covariates were selected based on prior literature and biological plausibility [12]. A detailed list of the variables and their inclusion in the models (as categorical or linear variables) is shown in Supplemental Table S1.

### 2.3. Analytical Strategy Informed by the D-Health Trial

The analytical framework was adapted from our previous analyses of vitamin D supplementation and all-cause mortality using UK Biobank data [24], but was modified to focus on infection-related hospitalizations and to follow the design and follow-up time of the D-Health Trial.

**Figure 1:**
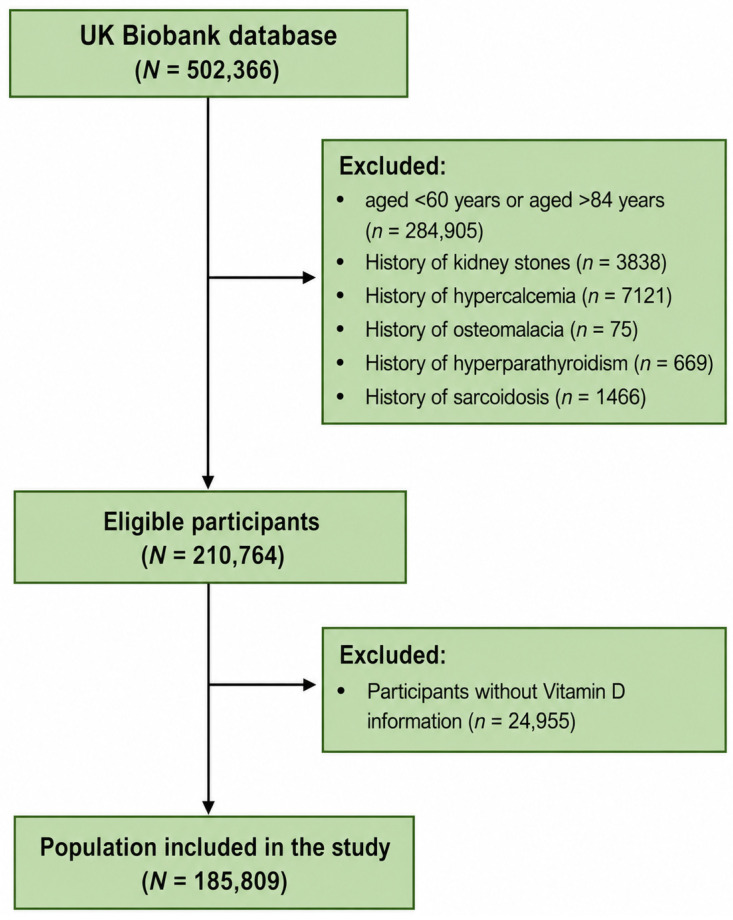
Flowcharts of UK Biobank participants selected using inclusion and exclusion criteria from the D-Health trial.

For this analysis, we employed a number of exclusion criteria that had been used in the D-Health Trial [23] to make the study populations as comparable as possible, as outlined in Figure 1. Participants were restricted to those aged 60–84 years, and individuals with a history of kidney stones, hypercalcemia, osteomalacia, hyperparathyroidism, or sarcoidosis were excluded. Participants without baseline serum 25(OH)D measurements were also excluded. After these exclusions, 185,809 participants were included in the analyses. Follow-up was censored at 5.7 years, corresponding to the median follow-up time of the D-Health Trial. We then conducted analyses to assess expected results of the D-Health Trial based on the distribution of baseline 25(OH)D concentrations. For that purpose, observations in the UKB study population were weighted to approximate the distribution of 25(OH)D concentrations observed in the D-Health Trial, as shown in Supplemental Table S2.

### 2.4. Statistical Analyses

Statistical analyses were performed using SAS statistical software (version 9.4, SAS Institute, Inc., Cary, NC, USA). All statistical tests were two-tailed, with a significance level of 0.05. Missing values for covariates were handled using multiple imputation under the assumption that data were missing at random. Five imputed datasets were generated and analysed separately, and the resulting estimates were combined using Rubin’s rules to account for uncertainty associated with missing data [29]. Analytical results from the imputed datasets were synthesized using the SAS procedure ‘PROC MIANALYZE’.

Negative binomial regression models, adjusted for the comprehensive list of covariates, were used to estimate incidence rate ratios (IRRs) and 95% confidence intervals (CIs) for the expected effect of a 38 nmol/L increase in 25(OH)D concentration on hospitalizations for infection. Additional subgroup analyses were conducted by sex and body mass index (BMI < 25 vs. ≥ 25 kg/m^2^). In the negative binomial regression analyses, the serum 25(OH)D concentration was treated as a continuous variable.

Subsequently, we repeated the analyses using an unweighted negative binomial regression analysis by restricting the study population to participants with baseline vitamin D insufficiency (<50 nmol/L) or deficiency (<30 nmol/L) to estimate the expected outcomes of targeted interventions among individuals with low vitamin D levels, as opposed to a non-targeted intervention.

Serum 25(OH)D concentrations are reported in nmol/L; conversion to ng/mL can be obtained by dividing by 2.5 (1 ng/mL = 2.5 nmol/L).

## 3. Results

### 3.1. Characteristics of the Study Population

Table 1 summarizes the baseline characteristics of the UKB participants who met the inclusion criteria of the D-Health trial. A total of 185,809 participants were included. The mean age at baseline was 64.1 years (SD 2.9), and 51.2% of participants were female. The mean baseline serum 25(OH)D concentration was 50.9 nmol/L. Overall, 31,510 participants (17.0%) had 25(OH)D concentrations below 30 nmol/L, 62,182 (33.5%) had concentrations between 30 and <50 nmol/L, and 92,117 (49.6%) had concentrations ≥ 50 nmol/L.

Participants with lower baseline 25(OH)D concentrations differed in several characteristics from those with higher concentrations. They were more often obese, current smokers, and abstainers from alcohol. For example, obesity (BMI ≥ 30 kg/m^2^) was present in 34.9% of participants with 25(OH)D < 30 nmol/L compared with 19.2% among those with concentrations ≥ 50 nmol/L. Current smoking was also more common among participants with 25(OH)D < 30 nmol/L (13.5%) than among those with concentrations ≥ 50 nmol/L (6.3%), and alcohol consumption was more frequent in the lowest vitamin D category. Participants with lower baseline 25(OH)D concentrations differed significantly from those with higher concentrations with respect to smoking status, BMI, and alcohol consumption (all *p* < 0.001).

The distribution of all baseline characteristics of the UKB study participants, overall and by baseline 25(OH)D concentrations, is shown in Supplemental Table S1.

During follow-up, a total of 7741 hospitalizations for infection were recorded, including 3550 respiratory tract infections, 1360 gastrointestinal infections, and 1383 skin infections.

### 3.2. Comparison of 25(OH)D Distributions of the UKB and D-Health Trial Populations

**Figure 2:**
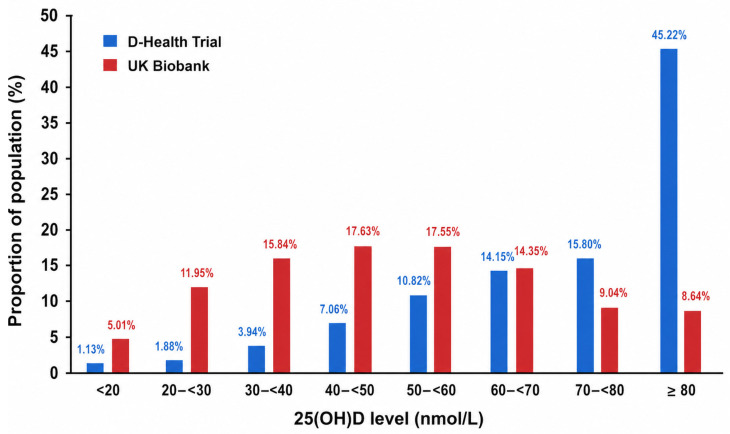
Comparison of baseline 25(OH)D distributions between the D-Health trial and matched cohort. The data of the D-Health Trial were derived from the mean (77 nmol/L) and standard deviation (25 nmol/L) in the placebo group reported by Neale et al. [28], assuming a normal distribution of 25(OH)D and using the “pnorm()” function in R software (version 4.4.1; R Foundation for Statistical Computing, Vienna, Austria).

Figure 2 compares 25(OH)D distributions across study populations. The D-Health Trial participants included a much higher proportion of participants with baseline 25(OH)D concentrations ≥ 50 nmol/L (86.0%), and much lower proportions of participants with vitamin D insufficiency or deficiency (14.0%) than the D-Health-type UKB cohort (50.4%).

### 3.3. Expected Impact of Increases in 25(OH)D Achieved in the Trial

Table 2 presents the estimated effects of a 38 nmol/L increase in serum 25(OH)D concentration on hospitalization for infection across different types. In the original D-Health trial, no statistically significant associations were observed for hospitalization for infection or for specific infection types.

Consistent with these findings, in the analyses in which study participants were weighted to match the baseline 25(OH)D distribution of the D-Health Trial (mean 77 nmol/L), no statistically significant protective effects were observed. The IRR for hospitalization for any infection was 1.08 (95% CI, 0.99–1.17), with similar null associations for respiratory tract infection (IRR 1.12, 95% CI 0.99–1.25), gastrointestinal infection (IRR 1.07, 95% CI 0.88–1.28), and skin infection (IRR 0.88, 95% CI 0.71–1.09).

In contrast, when the analyses were restricted to participants with lower baseline vitamin D status, consistent and statistically significant inverse associations were observed. Among participants with baseline 25(OH)D concentrations <50 nmol/L, a 38 nmol/L increase in 25(OH)D was associated with a significantly lower risk of hospitalization for any infection (IRR 0.85, 95% CI 0.80–0.90). Protective associations were also obtained for respiratory tract infection (IRR 0.84, 95% CI 0.77–0.91), gastrointestinal infection (IRR 0.79, 95% CI 0.70–0.91), and skin infection (IRR 0.76, 95% CI 0.67–0.87). These associations were further strengthened when restricting the study population to participants with baseline 25(OH)D concentrations <30 nmol/L. In this group, an increase of 25(OH)D levels by 38 nmol/L was associated with a 21% lower risk of hospitalization for any infection (IRR 0.79, 95% CI 0.73–0.86), with similarly strong inverse associations for hospitalizations for respiratory tract infection (IRR 0.76, 95% CI 0.68–0.85), gastrointestinal infection (IRR 0.74, 95% CI 0.62–0.88), and skin infection (IRR 0.78, 95% CI 0.66–0.92).

### 3.4. Subgroup Analyses by Sex and Body Mass Index

Subgroup analyses on hospitalizations for any type of infection stratified by sex and BMI are shown in Table 3. In the original D-health trial, no statistically significant associations were reported among either males or females, or across BMI strata.

In the “D-Health weighted analyses”, associations remained null among males (IRR 0.94, 95% CI 0.84–1.05) and females (IRR 1.10, 95% CI 0.98–1.23). Also, no statistically significant associations were observed across BMI categories in the weighted analyses (IRR 1.07, 95% CI 0.92–1.25 for BMI < 25 kg/m^2^; IRR 1.05, 95% CI 0.95–1.16 for BMI ≥ 25 kg/m^2^). In contrast, in analyses restricted to participants with a lower baseline vitamin D status, consistent inverse associations were observed across sex and BMI subgroups. Among participants with baseline 25(OH)D < 50 nmol/L, IRRs for hospitalization for infection were 0.73 (95% CI 0.67–0.80) in males and 0.84 (95% CI 0.77–0.92) in females. Similar protective associations were observed in both BMI strata (IRR 0.73, 95% CI 0.64–0.82 for BMI < 25 kg/m^2^; IRR 0.83, 95% CI 0.77–0.89 for BMI ≥ 25 kg/m^2^). Among participants with baseline 25(OH)D < 30 nmol/L, inverse associations were further strengthened, with IRRs of 0.69 (95% CI 0.61–0.77) in males and 0.77 (95% CI 0.68–0.85) in females. Corresponding IRRs were 0.67 (95% CI 0.57–0.79) for BMI < 25 kg/m^2^ and 0.76 (95% CI 0.69–0.83) for BMI ≥ 25 kg/m^2^.

Additional analyses using BMI < 30 and ≥30 kg/m^2^ yielded similar findings. In both BMI groups, higher 25(OH)D concentrations were associated with lower risks of hospitalization for infection among participants with vitamin D insufficiency or deficiency, although the associations were somewhat weaker among individuals with obesity (BMI ≥ 30 kg/m^2^) (Supplementary Table S3).

## 4. Discussion

In these analyses based on a subpopulation of the UK Biobank that met the D-Health Trial inclusion criteria, increases in serum 25(OH)D concentrations comparable to those achieved in the D-Health Trial were not associated with meaningful reductions in hospitalization for infection when the analyses reflected the baseline vitamin D distribution of the original trial population. In contrast, when the analyses were restricted to individuals with baseline vitamin D insufficiency (25(OH)D concentrations < 50 nmol/L) or deficiency (25(OH)D concentrations < 30 nmol/L), substantial inverse associations were observed. Among participants with vitamin D insufficiency, a 38 nmol/L increase in 25(OH)D was associated with a 15% lower risk of hospitalization for any infection (IRR 0.85, 95% CI 0.80–0.90). Protective associations were also seen for respiratory infections (IRR 0.84, 95% CI 0.77–0.91), gastrointestinal infections (IRR 0.79, 95% CI 0.70–0.91), and skin infections (IRR 0.76, 95% CI 0.67–0.87). These associations were even stronger among participants with vitamin D deficiency, in whom a 38 nmol/L increase in 25(OH)D concentration was associated with a 21% lower risk of hospitalization for any infection (IRR 0.79, 95% CI 0.73–0.86).

Our findings are consistent with a large body of epidemiological evidence suggesting that low circulating 25(OH)D concentrations increase susceptibility to infections and infection-related hospitalizations. For example, a cohort study of 1713 Medicare beneficiaries in the US reported that older adults with serum 25(OH)D concentrations below 37.5 nmol/L (mean age 69.5 years) had a higher risk of hospitalization with an infection than those with higher concentrations (risk ratio 2.8; 95% CI 1.3–5.9) [12]. A previous study from Canada reported that 3–15-year-old children with 25(OH)D concentrations < 50 nmol/L had a 70% higher risk of laboratory-confirmed viral RTIs (hazard ratio, 1.67; 95% CI, 1.16–2.40) [30]. Furthermore, a recent study including a subsample of 36,258 UKB participants from different ethnic groups found that serum 25(OH)D status < 15 nmol/L was associated with a 33% higher HR for RTI hospitalization, compared with ≥75 nmol/L [31]. Similarly, a meta-analysis of observational studies found that low vitamin D status (serum 25(OH)D < 50 nmol/L) was associated with more severe COVID-19 outcomes, including increased risks of hospitalization (pooled odds ratio [OR] 2.38; 95% CI 1.56–3.63) and intensive care unit admission (pooled OR 2.16; 95% CI 1.43–3.26) [32]. The substantial risk reductions among participants with vitamin D insufficiency or deficiency observed in our study are therefore consistent with prior epidemiological evidence and with established biological mechanisms [33], and suggest that vitamin D may play an important role in preventing severe infection among individuals with low baseline concentrations.

The absence of an overall protective effect in the weighted analyses reflecting the baseline 25(OH)D concentrations of the D-Health Trial population closely mirrors the findings of the D-Health Trial [28], which reported little or no effect of monthly vitamin D supplementation on hospitalization for any infection (IRR: 0.95; 95% CI 0.86–1.05) in a largely vitamin D-replete population. Previous analyses of the D-Health trial also reported no effect of vitamin D supplementation on the risk of acute RTI (OR 0.98; 95% CI 0.93–1.02) [34]. In the D-Health Trial, the mean baseline 25(OH)D concentration was approximately 77 nmol/L, and only 14% of participants had concentrations below 50 nmol/L, limiting the ability to detect benefits confined to deficient individuals.

Other randomized trials of vitamin D supplementation for infection-related outcomes have likewise reported largely null results. A meta-analysis of RCTs published up to 1 May 2020 (19 studies, *n* = 21,813 participants) found vitamin D supplementation to be associated with a modest, but not significant reduction in respiratory infections requiring hospital admission or emergency department visits (OR: 0.90; 95% CI: 0.71, 1.14) [20]. A modest, albeit not statistically significant reduction in acute respiratory infections was likewise found in a meta-analysis of 30 RCTs involving 30,263 participants (relative risk 0.96; 95% CI 0.91–1.01) [21]. These meta-analyses of studies including participants with a broad range of baseline vitamin D statuses are consistent with the weighted results of the present study. By contrast, our analyses restricted to individuals with low baseline 25(OH)D concentrations suggest that clinically meaningful reductions in infection-related hospitalization may be expected in populations with vitamin D insufficiency or deficiency, highlighting the importance of baseline vitamin D status as a key determinant of potential benefit.

These findings are also consistent with our recently published findings on all-cause mortality using the same UK Biobank resource and an analogous analytical framework, in which null results were observed when analyses reflected the vitamin D-replete VITAL and D-Health trial populations, whereas substantially stronger effects were found among individuals with vitamin D insufficiency or deficiency [24]. These findings underline the importance of considering baseline vitamin D status in vitamin D supplementation and in interpreting vitamin D supplementation trial results.

In this study, the effects of increases in serum 25(OH)D concentrations were observed not only for overall infection-related hospitalizations but also for hospitalizations due to respiratory, gastrointestinal, and skin infections. The consistency of findings across infection types suggests that vitamin D may exert broad effects on host immune defence rather than acting on specific pathogens. Experimental evidence provides biological support for these findings and may help explain why stronger associations were observed among participants with vitamin D insufficiency or deficiency [35,36,37]. The stronger inverse associations observed among participants with vitamin D insufficiency or deficiency are biologically plausible. Vitamin D enhances innate immune defense by inducing antimicrobial peptides such as cathelicidin (LL-37) and β-defensins, which exhibit broad antibacterial, antiviral, and antifungal activity [35]. In addition, vitamin D promotes macrophage function, supports epithelial barrier integrity, and modulates inflammatory responses by reducing excessive production of pro-inflammatory cytokines while maintaining effective pathogen clearance [35,36]. Individuals with vitamin D deficiency may have impaired activation of these pathways, resulting in increased susceptibility to severe infections [36,37]. Correction of vitamin D deficiency may therefore restore immune competence and lead to greater clinical benefits than those expected among individuals who already have sufficient vitamin D concentrations. These mechanisms may explain why the strongest associations in our study were observed among participants with vitamin D deficiency (<30 nmol/L), who may derive the greatest immunological benefit from increases in circulating 25(OH)D concentrations.

Our findings are also consistent with the individual participant data meta-analysis by Martineau et al. [19], which pooled data from 25 randomized controlled trials of vitamin D supplementation and reported that protective effects against acute respiratory tract infections were strongest among participants with a low baseline vitamin D status, particularly those with 25(OH)D concentrations below 25 nmol/L. These findings support the hypothesis that baseline vitamin D status is an important determinant of responsiveness to vitamin D supplementation and may help explain why large trials conducted predominantly among vitamin D-replete populations, including the D-Health Trial, have generally reported null findings. The consistency between the Martineau meta-analysis [19] and our findings strengthens the interpretation that vitamin D-related benefits are most likely to occur among individuals with vitamin D insufficiency or deficiency.

In subgroup analyses, the clinical effects of increased 25(OH)D concentration appeared somewhat stronger in men and in individuals with lower BMI. These patterns are biologically plausible and consistent with known differences in vitamin D metabolism and distribution. Individuals with higher adiposity tend to have lower circulating 25(OH)D concentrations due to volumetric dilution and sequestration of vitamin D in adipose tissue, which may reduce the effective increase in circulating vitamin D following supplementation [38,39,40]. Consequently, a given increase in 25(OH)D concentration may translate into relatively greater biological effects among individuals with lower BMI.

Sex-related differences in vitamin D metabolism and immune function may also contribute. The stronger associations observed among men are consistent with known sex differences in immune responses and vitamin D metabolism reported in previous studies [41,42]. Experimental and clinical studies have shown that women generally exhibit stronger innate and adaptive immune responses than men, whereas men are more prone to dysregulated inflammatory responses during infection [41]. Sex-related differences in vitamin D status and vitamin D-associated inflammatory pathways have also been reported [42]. These biological differences may influence the immunomodulatory effects of vitamin D and could contribute to the stronger associations observed among men in our study, although the underlying mechanisms remain incompletely understood.

Our findings have several important implications for clinical practice and public health. First, they suggest that the lack of benefit observed in randomized trials of vitamin D supplementation may largely reflect the relatively high baseline vitamin D status of trial populations rather than an absence of causal effects. In populations where most individuals already have sufficient vitamin D concentrations, additional supplementation may provide limited incremental benefit for infection prevention. Second, our results support a more targeted approach to vitamin D supplementation. Rather than universal supplementation in generally vitamin D-replete populations, interventions focused on individuals with vitamin D insufficiency or deficiency may yield greater benefits in terms of reducing severe infection outcomes. Such targeted strategies may be particularly relevant for older adults, individuals with limited sun exposure, and populations with high prevalence of vitamin D deficiency. Third, given the substantial global burden of infection-related hospitalizations and the relatively low cost and safety profile of vitamin D supplementation, strategies aimed at correcting vitamin D deficiency could have meaningful public health impact. Our findings suggest that improving vitamin D status in deficient populations may reduce the risk of severe infections requiring hospitalization, thereby potentially reducing healthcare utilization and associated costs.

### Strengths and Limitations

Our study has several strengths. A major strength of this study is that our analyses were based on data from a very large cohort study, employing exclusion criteria, increases in 25(OH)D and follow-up time that closely matched those of the D-Health Trial, which, to date, is by far the largest clinical trial investigating the impact of vitamin D supplementation on hospitalizations due to infection. This approach provides a structured way to address the apparent differences between observational evidence and randomized trial findings. Use of the large, well-characterized prospective UKB cohort with detailed baseline data and linkage to hospital admission records allowed for robust assessment of infection-related hospitalizations. Furthermore, by modeling changes in 25(OH)D concentrations corresponding to those achieved in the randomized trial, our analyses directly address clinically relevant intervention effects.

Our study also has several limitations. First, although we approximated key aspects of the D-Health trial design and adjusted for a comprehensive list of potential confounders, residual confounding inherent to observational data cannot be entirely excluded. Second, the UK Biobank is not fully representative of the general population and may include relatively healthier volunteers, potentially limiting the generalizability of our findings. Moreover, differences in baseline vitamin D status, lifestyle patterns, healthcare systems, and environmental exposures across countries may influence the risk of infection-related hospitalization and reduce direct comparability with the original trials. Third, despite efforts to align age and sex distributions with those of the D-Health Trial, minor differences may remain and could have affected cross-study comparisons. In addition, given the restricted age range of the UKB population, we were unable to include age groups from 70–84 years that had been included in the D-Health trial. Furthermore, participants were recruited from the United Kingdom, a high-latitude region with lower levels of sunlight exposure than many countries, including most European countries. Consequently, the observed associations may not be directly generalizable to populations living in regions with substantially greater ultraviolet exposure, where determinants of vitamin D status may differ.

Despite these limitations, our findings support the importance of targeting vitamin D supplementation to individuals with low baseline vitamin D concentrations. Future randomized trials designed to evaluate the health effects of vitamin D supplementation may benefit from focusing on populations with vitamin D insufficiency or deficiency.

## 5. Conclusions

In these analyses of UK Biobank data, increases in serum 25-hydroxyvitamin D concentrations comparable to those achieved in the D-Health Trial were not associated with reductions in hospitalization for infection when analyses reflected the baseline vitamin D distribution of the original trial population. However, substantial reductions in infection-related hospitalizations were observed when the analyses were restricted to individuals with vitamin D insufficiency or deficiency. These findings suggest that the null results of the D-Health Trial, the largest trial reporting on this outcome to date, may be explained by its conduction in a predominantly vitamin D-replete population and that clinically relevant benefits of supplementation might be expected in populations with low baseline 25(OH)D concentrations. Future randomized trials evaluating the potential role of vitamin D supplementation in infection prevention should specifically target individuals with insufficient or deficient vitamin D concentrations, among whom the strongest inverse associations were observed in the present study.

## Figures and Tables

**Table 1:** Characteristics of the study population.

Characteristic	All (*n* = 185,809)	25(OH)D <30 nmol/L (*n* = 31,510)	30–<50 nmol/L (*n* = 62,182)	≥50 nmol/L (*n* = 92,117)
Sex, No. (%)				
Female	95,068 (51.2)	16,456 (52.2)	32,325 (52.0)	46,287 (50.3)
Male	90,741 (48.8)	15,054 (47.8)	29,857 (48.0)	45,830 (49.8)
Age (years), mean (SD)	64.1 (2.9)	64.0 (2.9)	64.1 (2.9)	64.2 (2.8)
Deprivation index at recruitment (points), median (IQR)	−0.4 (−0.7; 0.3)	−0.2 (−0.6;0.7)	−0.4 (−0.7; 0.3)	−0.5 (−0.8; 0.1)
School education, No. (%)				
≤9 years	64,267 (34.6)	11,174 (35.5)	20,953 (33.7)	32,140 (34.9)
10–11 years	47,782 (25.7)	7271 (23.1)	15,602 (25.1)	24,909 (27.0)
≥12 years	73,760 (39.7)	13,065 (41.5)	25,627 (41.2)	35,608 (38.1)
IPAQ activity group, *n* (%)				
Low	23,082 (16.6)	5470 (24.4)	8326 (18.0)	9286 (13.1)
Moderate	57,852 (41.5)	9691 (43.1)	19,880 (43.0)	28,281 (40.0)
High	58,442 (41.9)	7306 (32.5)	17,996 (39.0)	33,140 (46.9)
BMI, No. (%)				
<18.5 kg/m^2^	794 (0.4)	168 (0.5)	223 (0.4)	403 (0.4)
18.5–<25 kg/m^2^	54,543 (29.5)	7461 (23.9)	16,052 (25.9)	31,030 (33.8)
25–<30 kg/m^2^	83,584 (45.2)	12,736 (40.7)	28,062 (45.3)	42,786 (46.6)
≥30 kg/m^2^	46,171 (24.9)	10,897 (34.9)	17,616 (28.4)	17,658 (19.2)
Smoking, No. (%)				
Never	92,749 (49.9)	14,796 (47.0)	31,166 (50.1)	46,787 (50.8)
Former	77,837 (41.9)	12,453 (39.5)	25,914 (41.7)	39,470 (42.9)
Current	15,174 (8.2)	4247 (13.5)	5088 (8.2)	5839 (6.3)
Alcohol consumption, No. (%)				
Abstainer	57,280 (30.8)	12,562 (39.9)	20,239 (32.6)	24,479 (26.6)
Women 0–19.99, men 0–39.99 g/d	76,239 (41.0)	11,089 (35.2)	25,135 (40.4)	40,015 (43.4)
Women 20–39.99, men 40–59.99 g/d	30,770 (16.6)	4230 (13.4)	9911 (15.9)	16,629 (18.1)
Women ≥ 40 g/d, men ≥ 60 g/d	21,520 (11.6)	3629 (11.5)	6897 (11.1)	10,994 (11.9)
25(OH)D (nmol/L), median (IQR)	49.7 (35.2; 64.5)	23.3 (19.0; 26.9)	40.5 (35.4; 45.2)	64.6 (56.9; 75.1)
Hospitalizations during follow-up, *n* episodes				
For any infection, No. (%)	7741 (4.2)	1770 (5.6)	2620 (4.2)	3351 (3.6)
For respiratory tract infection, No. (%)	3550 (1.9)	821 (2.6)	1218 (2.0)	1511 (1.6)
For gastrointestinal infection, No. (%)	1360 (0.7)	306 (1.0)	469 (0.8)	585 (0.6)
For skin infection, No. (%)	1383 (0.7)	334 (1.1)	495 (0.8)	554 (0.6)

SD = Standard Deviation; IQR = Interquartile Range; BMI = Body Mass Index; 25(OH)D = 25-hydroxy-vitamin D. Note: Numbers may not add up to total due to missing values.

**Table 2:** Estimated effects of a 38 nmol/L increase in 25-hydroxy-vitamin D concentration on hospitalization for infection.

	Baseline 25(OH)D		Incidence Rate Ratios (95% CI)			
	Inclusion Criteria	Mean	All (*n* Episodes = 7741)	Respiratory Tract Infection (*n* Episodes = 3550)	Gastrointestinal Infection (*n* Episodes = 1360)	Skin Infection (*n* Episodes = 1383)
D-Health, reported	Unrestricted	77 nmol/L	0.95 (0.86–1.05)	0.93 (0.81–1.08)	1.03 (0.84–1.26)	0.95 (0.76–1.20)
UKB cohort	Unrestricted, weighted *	77 nmol/L	1.08 (0.99–1.17)	1.12 (0.99–1.25)	1.07 (0.88–1.28)	0.88 (0.71–1.09)
	<50 nmol/L	34 nmol/L	0.85 (0.80–0.90)	0.84 (0.77–0.91)	0.79 (0.70–0.91)	0.76 (0.67–0.87)
	<30 nmol/L	23 nmol/L	0.79 (0.73–0.86)	0.76 (0.68–0.85)	0.74 (0.62–0.88)	0.78 (0.66–0.92)

* Weighted to yield the same 25(OH)D distribution as observed in the D-Health trial.

**Table 3:** Estimated effects of a 38 nmol/L increase in 25-hydroxy-vitamin D concentration on hospitalization for any infection in subgroups of participants by sex and BMI.

	Baseline 25(OH)D		Incidence Rate Ratios (95% CI) by Sex		Incidence Rate Ratios (95% CI) by BMI	
	Inclusion Criteria	Mean	Males	Females	BMI < 25 kg/m^2^	BMI ≥ 25 kg/m^2^
D-Health, reported	Unrestricted	77 nmol/L	0.95 (0.83–1.09)	0.94 (0.80–1.11)	1.07 (0.88–1.31)	0.92 (0.81–1.03)
UKB cohort	Unrestricted, weighted *	77 nmol/L	0.94 (0.84–1.05)	1.10 (0.98–1.23)	1.07 (0.92–1.25)	1.05 (0.95–1.16)
	<50 nmol/L	34 nmol/L	0.73 (0.67–0.80)	0.84 (0.77–0.92)	0.73 (0.64–0.82)	0.83 (0.77–0.89)
	<30 nmol/L	23 nmol/L	0.69 (0.61–0.77)	0.77 (0.68–0.85)	0.67 (0.57–0.79)	0.76 (0.69–0.83)

* Weighted to yield the same 25(OH)D distribution as observed in the D-Health trial.

## Data Availability

This study utilized data from the UK Biobank Resource, obtained under application number “89329”. Publicly available data is accessible to researchers via an open application on https://www.ukbiobank.ac.uk/register-apply/, accessed on 10 July 2026.

## Supplementary Materials

The following supporting information can be downloaded at: https://www.mdpi.com/article/10.3390/nu18142276/s1. Table S1. Distribution of baseline characteristics of the study population, overall and by serum 25(OH)D concentration; Table S2. Weighting of observations in UK Biobank to approximate the same distribution of vitamin D concentrations as observed in the D-Health Trial; Table S3. Estimated effects of a 38 nmol/L increase in 25-hydroxy-vitamin D concentration on hospitalization for any infection in subgroups of participants by BMI.
