# Supplementary material for: Impact of Vitamin D Supplementation on Hospitalizations for Infection: Results of the D-Health Trial Revisited
Source: Nutrients. 2026 Jul 11;18(14):2276. doi: 10.3390/nu18142276 (PMC13414773; doi:10.3390/nu18142276)
Supplement: Supplementary file 1 [file nutrients-18-02276-s001.zip › nutrients-4354592-supplementary.pdf]

Supplemental Materials

Table of Contents

Supplemental Table S1. Distribution of baseline characteristics of the study population, overall and by serum 25(OH)D concentration 2

Supplemental Table S2. Weighting of observations in UK Biobank to approximate the same distribution of vitamin D concentrations as observed in the D-Health Trial 5

Supplemental Table S3. Estimated effects of a 38 nmol/L increase in 25-hydroxy-vitamin D concentration on hospitalization for any infection in subgroups of participants by BMI 6

Supplemental Table S1. Distribution of baseline characteristics of the study population, overall and by serum 25(OH)D concentration

Characteristic	All (n= 185 809)	25(OH)D < 30 nmol/L (n=31,510)	30-< 50 nmol/L (n= 62,182)	⩾ 50 nmol/L (n=92,117)
**SOCIO-DEMOGRAPHIC/-ECONOMIC FACTORS**				
**Age (years),** median (IQR)	64 (62; 66)	64 (61; 66)	64 (62; 66)	64 (62; 66)
**Sex,** n (%)				
Female	95 068 (51.2)	16 456 (52.2)	32 325 (52.0)	46 287 (50.3)
Male	90 741 (48.8)	15 054 (47.8)	29 857 (48.0)	45 830 (49.8)
**Education (years),** median (IQR)	10 (9; 17)	10 (9;17)	10 (9; 17)	10 (9; 17)
**Deprivation index at recruitment (points),** median (IQR)	-0.4 (-0.7; 0.3)	-0.2 (-0.6;0.7)	-0.4 (-0.7; 0.3)	-0.5 (-0.8; 0.1)
**No. of individuals in household,** n (%)				
1	39 261 (21.3)	8619 (27.7)	13 712 (22.3)	16 930 (18.5)
2	119 320 (64.8)	17 043 (54.8)	38 936 (63.2)	63 341 (69.2)
3	18 128 (9.8)	3577 (11.5)	6385 (10.4)	8166 (8.9)
4	5362 (2.9)	1253 (4.0)	1853 (3.0)	2256 (2.5)
5	1294 (0.7)	341 (1.0)	446 (0.7)	507 (0.6)
6	404 (0.2)	132 (0.4)	146 (0.2)	126 (0.1)
7	405 (0.2)	112 (0.4)	144 (0.2)	149 (0.2)
**Annual household income (£),** n (%)				
< 18,000	51 300 (33.9)	10 255 (39.9)	17 341 (34.1)	23 704 (31.7)
18,000 - < 30,999	49 356 (32.6)	7613 (29.7)	16 331 (32.1)	25 412 (34.0)
31,000- < 51,999	31 652 (20.9)	4774 (18.6)	10 680 (21.0)	16 198 (21.7)
52,000 - < 100,000	15 403 (10.2)	2487 (9.7)	5268 (10.4)	7648 (10.2)
≥ 100,000	3699 (2.4)	550 (2.1)	1286 (2.5)	1863 (2.5)
**LIFE-STYLE FACTORS**				
**Smoking,** n (%)				
Never	92 749 (49.9)	14 796 (47.0)	31 166 (50.1)	46 787 (50.8)
Former	77 837 (41.9)	12 453 (39.5)	25 914 (41.7)	39 470 (42.9)
Current	15 174 (8.2)	4247 (13.5)	5088 (8.2)	5839 (6.3)
**Alcohol consumption (g/d),** n (%)				
Abstainer	57 280 (30.8)	12 562 (39.9)	20 239 (32.6)	24 479 (26.6)
Women 0 - < 20 / men 0 - < 40	76 239 (41.0)	11 089 (35.2)	25 135 (40.4)	40 015 (43.4)
Women 20 - < 40 / men 40 - < 60	30 770 (16.6)	4230 (13.4)	9911 (15.9)	16629 (18.1)
Women ≥ 40 / men ≥ 60	21 520 (11.6)	3629 (11.5)	6897 (11.1)	10994 (11.9)
**IPAQ activity group,** n (%)				
Low	23 082 (16.6)	5470 (24.4)	8326 (18.0)	9286 (13.1)
Moderate	57 852 (41.5)	9691 (43.1)	19 880 (43.0)	28281 (40.0)
High	58 442 (41.9)	7306 (32.5)	17 996 (39.0)	33140 (46.9)
**Frequency of visiting friends/family,** n (%)				
Once every few months/rare	12 839 (7.0)	2952 (9.5)	4389 (7.1)	5498 (6.0)
Once/month	20 251 (11.0)	3706 (11.9)	6859 (11.1)	9686 (10.6)
Once/week	60 019 (32.5)	9919 (31.7)	19 935 (32.2)	30 165 (32.9)
2-4 times/week	64 855 (35.1)	10 148 (32.5)	21 506 (34.8)	33 201 (36.2)
Almost daily	26 779 (14.5)	4521 (14.5)	9148 (14.8)	13 110 (14.3)
**Oily fish consumption,** n (%)				
Never/ less than once a week	68 030 (36.8)	14 133 (45.2)	22 877 (37.0)	31 020 (33.8)
At least once a week	116 829 (63.2)	17 108 (54.8)	39 004 (63.0)	60 717 (66.2)
**Cereal consumption (bowls/week),** n (%)				
Never	28 128 (15.2)	6284 (20.0)	9577 (15.4)	12 267 (13.3)
< 7	71 849 (38.8)	12625 (40.3)	24 389 (39.3)	34 835 (37.9)

Characteristic	All (n= 185 809)	25(OH)D < 30 nmol/L (n=31,510)	30-< 50 nmol/L (n= 62,182)	⩾ 50 nmol/L (n=92,117)
≥ 7	85 361 (46.1)	12442 (39.7)	28 061 (45.2)	44 858 (48.8)
**Processed meat intake,** n (%)				
Never/ less than once a week	73 569 (39.7)	12 264 (39.1)	24 275 (39.1)	37 030 (40.3)
At least once a week	111 900 (60.3)	19 145 (61.0)	37 795 (60.9)	54 960 (59.8)
**Milk consumption,** n (%)				
Never/rarely	5703 (3.1)	1169 (3.7)	1937 (3.1)	2597 (2.8)
Occasionally/regularly	180 000 (96.9)	30 311 (96.3)	60 207 (96.9)	89482 (97.2)
**Spread consumption,** n (%)				
Never/rarely	18 987 (10.2)	3250 (10.3)	6059 (9.8)	9678 (10.5)
Butter	64 518 (34.8)	13 187 (41.9)	21 726 (35.0)	29 605 (32.2)
Margarine/others	102 069 (55.0)	15 009 (47.7)	34 319 (55.3)	52 741 (57.3)
**Preferred bread type,** n (%)				
White	45 333 (25.1)	9436 (30.8)	15 330 (25.4)	20 567 (23.1)
Brown	22 775 (12.7)	4057 (13.3)	7661 (12.7)	11 057 (12.4)
Wholemeal or wholegrain	104 382 (58.0)	15 755 (51.5)	34 829 (57.7)	53 798 (60.4)
Other type of bread	7647 (4.3)	1346 (4.4)	2593 (4.3)	3708 (4.2)
**DISEASES & DISEASE SYMPTOMS**				
**Diabetes,** n (%)				
No	173 035 (93.1)	28 179 (89.5)	57 634 (92.7)	87 222 (94.7)
Yes	12 739 (6.9)	3320 (10.5)	4536 (7.3)	4883 (5.3)
**COPD,** n (%)				
No	184 750 (99.5)	31 226 (99.1)	61 797 (99.4)	91 727 (99.6)
Yes	1024 (0.6)	273 (0.9)	373 (0.6)	378 (0.4)
**CHD,** n (%)				
No	171 025 (92.1)	28 394 (90.1)	57 122 (91.9)	85 509 (92.8)
Yes	14 749 (7.9)	3 105 (9.9)	5048 (8.1)	6596 (7.2)
**Stroke,** n (%)				
No	181 933 (97.9)	30 555 (97.0)	60 881 (97.9)	90 497 (98.3)
Yes	3841 (2.1)	944 (3.0)	1289 (2.1)	1608 (1.7)
**Number of self-reported chronic diseases,** median (IQR)	2 (1-3)	2 (1-4)	2 (1-3)	2 (1-3)
**Number of treatments/medications taken,** median (IQR)	3 (1-5)	3 (1-5)	2 (1-5)	3 (1-5)
**BIOMARKERS**				
**BMI (kg/m²),** n (%)				
< 18.5 kg/m²	794 (0.4)	168 (0.5)	223 (0.4)	403 (0.4)
18.5 - <25 kg/m²	54 543 (29.5)	7461 (23.9)	16 052 (25.9)	31 030 (33.8)
25 - < 30 kg/m²	83 584 (45.2)	12 736 (40.7)	28 062 (45.3)	42 786 (46.6)
≥30 kg/m²	46 171 (24.9)	10 897 (34.9)	17 616 (28.4)	17 658 (19.2)
**VITAMIN D SPECIFIC FACTORS**				
**25(OH)D level (nmol/L),** median (IQR)	49.7 (35.2; 64.5)	23.3 (19.0; 26.9)	40.5 (35.4; 45.2)	64.6 (56.9; 75.1)
**Latitude of study center (per 1°),** median (IQR)	53.4 (51.5; 53.8)	53.4 (51.5; 53.8)	53.4 (51.5; 53.8)	53.0 (51.5; 53.8)
**Month of attending the study center (month of blood draw),** n (%)				
1	12 476 (6.7)	3656 (11.6)	4783 (7.7)	4037 (4.4)
2	14 686 (7.9)	4716 (15.0)	5384 (8.7)	4586 (5.0)
3	18 314 (9.9)	5729 (18.2)	6594 (10.6)	5991 (6.5)
4	16 320 (8.8)	4359 (13.8)	6128 (9.9)	5833 (6.3)

Characteristic	All (n= 185 809)	25(OH)D < 30 nmol/L (n=31,510)	30-< 50 nmol/L (n= 62,182)	≥ 50 nmol/L (n=92,117)
5	19 104 (10.3)	3205 (10.2)	7360 (11.8)	8539 (9.3)
6	19 429 (10.5)	1550 (4.9)	6184 (10.0)	11 695 (12.7)
7	16 259 (8.8)	900 (2.9)	4101 (6.6)	11 258 (12.2)
8	14 243 (7.7)	668 (2.1)	3482 (5.6)	10 093 (11.0)
9	13 155 (7.1)	729 (2.3)	3519 (5.7)	8907 (9.7)
10	16 119 (8.7)	1431 (4.5)	4995 (8.0)	9693 (10.5)
11	15 493 (8.3)	2471 (7.8)	5684 (9.1)	7338 (8.0)
12	10 211 (5.5)	2096 (6.7)	3968 (6.4)	4147 (4.5)
**Time spent outdoors in summer (h/day),** n (%)				
<1	5156 (2.9)	1633 (5.7)	1941 (3.3)	1582 (1.8)
1-2	39 214 (22.4)	8109 (28.1)	14 227 (24.4)	16 878 (19.2)
3-4	60 957 (34.8)	10 036 (34.7)	20 457 (35.0)	30 464 (34.7)
5-6	45 956 (26.2)	6126 (21.2)	14 521 (24.9)	25 309 (28.8)
≥ 7	23 879 (13.6)	2992 (10.4)	7241 (12.4)	13 646 (15.5)
**Time spent outdoors in winter (h/day),** n (%)				
<1	25 396 (14.6)	6043 (20.8)	9038 (15.5)	10 315 (11.8)
1-2	99 066 (56.8)	16 223 (56.0)	33 576 (57.7)	49 267 (56.4)
3-4	34 535 (19.8)	4652 (16.0)	10 779 (18.5)	19 104 (21.9)
5-6	10 781 (6.2)	1395 (4.8)	3282 (5.6)	6104 (7.0)
≥ 7	4723 (2.7)	684 (2.4)	1511 (2.6)	2528 (2.9)
**Skin color,** n (%)				
Very fair	13 259 (7.2)	2872 (9.3)	4874 (8.0)	5513 (6.1)
Fair	131 504 (71.8)	21 769 (70.3)	44 368 (72.4)	65 367 (72.0)
Light olive	31 137 (17.0)	4316 (13.9)	9723 (15.9)	17 098 (18.8)
Dark olive	3006 (1.6)	446 (1.4)	910 (1.5)	1650 (1.8)
Brown	3402 (1.9)	1273 (4.1)	1111 (1.8)	1018 (1.1)
Black	748 (0.4)	275 (0.9)	296 (0.5)	177 (0.2)
**Ease of skin tanning,** n (%)				
Very tanned	39 401 (21.8)	5456 (18.0)	11 994 (19.8)	21 951 (24.3)
Moderately tanned	74 045 (40.9)	10 748 (35.5)	23 919 (39.5)	39 378 (43.7)
Mildly/occasionally tanned	35 831 (19.8)	6938 (22.9)	12 988 (21.5)	15 905 (17.6)
Never tan, only burn	31 749 (17.5)	7130 (23.6)	11 639 (19.2)	12 980 (14.4)
**Sun screen/UV protection use,** n (%)				
Never/rarely	19 678 (10.6)	5280 (16.8)	6826 (11.0)	7572 (8.2)
Sometimes	63 895 (34.4)	10 907 (34.7)	21 746 (35.0)	31 242 (33.9)
Most of times	62 456 (33.7)	8778 (27.9)	20 666 (33.3)	33 012 (35.9)
Always	38 391 (20.7)	5957 (19.0)	12 458 (20.1)	19 976 (21.7)
Do not go out in sunshine	1187 (0.6)	501 (1.6)	420 (0.7)	266 (0.3)
**Solarium/sunlamp use (times per year),** n (%)				
Never	172 565 (93.7)	29 432 (94.8)	58 203 (94.4)	84 930 (92.8)
< 1	7890 (4.3)	1421 (4.6)	2725 (4.4)	3744 (4.1)
1 - 6	2162 (1.2)	153 (0.5)	522 (0.9)	1487 (1.6)
7 - 12	847 (0.5)	32 (0.1)	130 (0.2)	685 (0.8)
> 12	783 (0.4)	13 (0.04)	89 (0.1)	681 (0.7)

Abbreviations: IQR: Interquartile Range, IPAQ: International Physical Activity Questionnaire, CHD: coronary heart disease, BMI: Body Mass Index, 25(OH)D: 25-hydroxy-vitamin D, UV: Ultraviolet.

Note: Numbers may not add up to total because of missing values.

Supplemental Table S2. Weighting of observations in UK Biobank to approximate the same distribution of vitamin D concentrations as observed in the D-Health Trial

25(OH)D [nmol/L]	Proportion of participants [%]		Weight (a/b)
	D-Health Trial (a)[1]	UK Biobank (b)	
**<20**	1.13	5.01	0.226
**20-<30**	1.88	11.95	0.157
**30-<40**	3.94	15.84	0.249
**40-<50**	7.06	17.63	0.400
**50-<60**	10.82	17.55	0.617
**60-<70**	14.15	14.35	0.986
**70-<80**	15.80	9.04	1.748
**80+**	45.22	8.64	5.234

Abbreviations: 25(OH)D: 25-hydroxy-vitamin D.

[1] Data were approximated from the mean (77 nmol/L) and standard deviation (25 nmol/L) in the placebo group reported by *Neale et al*, assuming normal distribution of 25(OH)D and using the "pnorm()" function in R software.

Supplemental Table S3. Estimated effects of a 38 nmol/L increase in 25-hydroxy-vitamin D concentration on hospitalization for any infection in subgroups of participants by BMI

	Baseline 25(OH)D		Incidence rate ratios (95% CI) by BMI	
	**Inclusion criteria**	**Mean**	**BMI<30 kg/m²**	**BMI≥30 kg/m²**
**UKB cohort**	Unrestricted, weighted*	77 nmol/L	1.10 (0.99-1.22)	1.00 (0.86-1.16)
	<50 nmol/L	34 nmol/L	0.82 (0.79-0.85)	0.90 (0.85-0.94)
	<30 nmol/L	23 nmol/L	0.77 (0.73-0.80)	0.83 (0.78-0.88)

*Weighted to yield the same 25(OH)D distribution as observed in the D-Health trial.
